# An Orally Active *Cannabis* Extract with High Content in Cannabidiol attenuates Chemically-induced Intestinal Inflammation and Hypermotility in the Mouse

**DOI:** 10.3389/fphar.2016.00341

**Published:** 2016-10-04

**Authors:** Ester Pagano, Raffaele Capasso, Fabiana Piscitelli, Barbara Romano, Olga A. Parisi, Stefania Finizio, Anna Lauritano, Vincenzo Di Marzo, Angelo A. Izzo, Francesca Borrelli

**Affiliations:** ^1^Department of Pharmacy, University of Naples Federico IINaples, Italy; ^2^Institute of Bimolecular Chemistry, ICB, National Research Council, PozzuoliItaly; ^3^Institute of Biomolecular Chemistry, Consiglio Nazionale delle RicerchePozzuoli, Italy

**Keywords:** *Cannabis sativa*, cannabidiol, cannabinoids, inflammatory bowel disease, colitis, intestinal motility

## Abstract

Anecdotal and scientific evidence suggests that *Cannabis* use may be beneficial in inflammatory bowel disease (IBD) patients. Here, we have investigated the effect of a standardized *Cannabis sativa* extract with high content of cannabidiol (CBD), here named CBD BDS for “CBD botanical drug substance,” on mucosal inflammation and hypermotility in mouse models of intestinal inflammation. Colitis was induced in mice by intracolonic administration of dinitrobenzenesulfonic acid (DNBS). Motility was evaluated in the experimental model of intestinal hypermotility induced by irritant croton oil. CBD BDS or pure CBD were given - either intraperitoneally or by oral gavage – after the inflammatory insult (curative protocol). The amounts of CBD in the colon, brain, and liver after the oral treatments were measured by high-performance liquid chromatography coupled to ion trap-time of flight mass spectrometry. CBD BDS, both when given intraperitoneally and by oral gavage, decreased the extent of the damage (as revealed by the decrease in the colon weight/length ratio and myeloperoxidase activity) in the DNBS model of colitis. It also reduced intestinal hypermotility (at doses lower than those required to affect transit in healthy mice) in the croton oil model of intestinal hypermotility. Under the same experimental conditions, pure CBD did not ameliorate colitis while it normalized croton oil-induced hypermotility when given intraperitoneally (in a dose-related fashion) or orally (only at one dose). In conclusion, CBD BDS, given after the inflammatory insult, attenuates injury and motility in intestinal models of inflammation. These findings sustain the rationale of combining CBD with other minor *Cannabis* constituents and support the clinical development of CBD BDS for IBD treatment.

## Introduction

Inflammatory bowel disease (IBD) is a a chronic immunologically mediated disease with growing incidence and prevalence rates in industrialized countries (Ananthakrishnan, 2015; Taleban et al., 2015). Effective pharmacotherapies for IBD are not always available and existing drugs may cause substantial side-effects leading to poor patient adherence. The anecdotal use of marijuana and/or preparations from *Cannabis sativa* L. (hemp) in IBD patients has been recently confirmed by investigations in humans (Ravikoff Allegretti et al., 2013; Naftali et al., 2014; Storr et al., 2014; Izzo et al., 2015). For example, a large-scale population based survey has recently suggested that IBD patients experience symptom relief with marijuana use (Weiss and Friedenberg, 2015). The *C. sativa* plant produces over 100 terpenophenolic molecules, i.e., the phytocannabinoids, which accumulates predominantly in the plant glandular trichomes (Russo, 2011). Clearly, the most known among the phytocannabinoids is Δ^9^-tetrahydrocannabinol (THC), whose possible clinical use is hindered by its psychoactivity. This obstacle has addressed further research toward non-psychotropic phytocannabinoids such as cannabidiol (CBD), the versatile pharmacology of which is well established (Esposito et al., 2013; Welty et al., 2014; Brodie et al., 2015; Burstein, 2015; McPartland et al., 2015).

Among numerous pharmacological actions of potential therapeutic interest, pure CBD has been shown to ameliorate experimental colitis (Borrelli et al., 2009; Jamontt et al., 2010; Schicho and Storr, 2012) and to normalize motility in the inflamed mouse gut (Capasso et al., 2008; Lin et al., 2011). It is worthy of note that the beneficial effects of pure CBD in the inflamed gut have been not observed/evaluated after oral gavage (intragastric) administration, a route of drug administration which is easy to be translated to humans for therapeutic use. In the past few years, the contribution of minor phytocannabinoids to the complex *Cannabis* pharmacology has been shown (Pertwee, 2008; Hill et al., 2012; McPartland and Russo, 2014) and a number of examples of additive/synergistic effects among the phytocannabinoids have been reported (Williamson and Evans, 2000; Wilkinson et al., 2003; DeLong et al., 2010; Jamontt et al., 2010; Russo, 2011; McPartland and Russo, 2014). This observation prompted the cultivation of specific *C. sativa* chemotypes (chemical genotypes) with high yields of a specific cannabinoid (Potter, 2014). To produce a *C. sativa* extract, the flowers of female plants are immersed in liquid carbon dioxide at extremely high pressure and the chemical compounds dissolving in this solvent are then separated and purified (Potter, 2014). One of the best studied among such extracts is the standardized *C. sativa* extract with high content of CBD, generally referred to as CBD BDS (an acronym for Botanical Drug Substance). Notably, CBD BDS is a major ingredient of the medicine known with the generic name nabiximols (Sativex^®^; GW Pharmaceuticals, Cambridge, UK), approved in many countries for the treatment of refractory spasticity in multiple sclerosis (Syed et al., 2014). In the present study, we have examined the effect of CBD BDS in the murine model of colitis induced by dinitrobenzenesulfonic acid. In addition, because motility alterations represent an hallmark in IBD patients, we also investigated the effect of the *C. sativa* extract in the model of intestinal dysmotility induced by the pro-inflammatory agent croton oil.

## Materials and Methods

### Drugs and Reagents

A standardized *C. sativa* extract with high content in CBD (CBD BDS, 63.9% w/w of CBD content) and pure CBD, [purity by high-performance liquid chromatography (HPLC), 99%] were supplied by GW Pharmaceuticals (Cambridge, UK). The dose of CBD BDS used in the experiments refers to the amount of CBD contained in the extract (e.g., 10 mg/kg of CBD BDS indicates a dose of the BDS that contains 10 mg/kg of CBD). 2,4,6-dinitrobenzenesulfonic acid (DNBS), croton oil and myeloperoxidase (MPO) from human leucocytes, were purchased from Sigma Aldrich S.r.l. (Milan, Italy). Pure CBD and CBD BDS were dissolved in ethanol/Tween20/saline (1:1:8) for i.p. injection (60 μl/mouse) or in sesame oil (90 μl/mouse) for oral gavage administration. DNBS was solubilized in 50% ethanol (0.15 ml/mouse). The pure CBD and CBD BDS vehicles had no significant effects on the responses under study.

### Plant Material Extraction and Composition of the *Cannabis* Extract

A *C. sativa* chemotype with a controlled high amount of CBD (de Meijer et al., 2003) was used for the preparation of CBD BDS. Details about the extraction, purification as well as typical HPLC chromatogram are reported elsewhere (Romano et al., 2014). The composition (%, w/w) of the main phytocannabinoids in CBD BDS was CBD 63.9 ± 5.9, Δ^9^-THC 3.0. cannabigerol 2.8, cannabichromene 3.1, cannabidivarin 1.4. The extract was prepared in GW Pharmaceuticals laboratories (Cambridge, UK).

### Animals

Male ICR mice, weighing 20–25 g for upper gastrointestinal transit experiments and 25–30 g for colitis experiments, were obtained from Charles River Laboratories (Calco, Lecco, Italy) and housed in polycarbonate cages under a 12 h light/dark cycle with light on at 07:00 a.m., controlled temperature (23 ± 2°C) and constant humidity (60%). Mice were fed *ad libitum* with standard food, except for the 24 h period immediately preceding the administration of DNBS, for the 12 h period preceding the measurement of intestinal transit and for the 2 h period preceding the oral gavage of drugs. All experiments complied with the Italian D.L. no. 116 of 27 January 1992 and associated guidelines in the European Communities Council Directive of 24 November 1986 (86/609/ECC). According to recent preclinical guidelines in pharmacology, group data subjected to statistical analysis had a minimum of *n* = 5 independent animals per group (Curtis et al., 2015).

### Induction of Experimental Colitis

Colitis was induced by the intracolonic administration of DNBS as described before (Borrelli et al., 2015). Briefly, mice were anesthetized with inhaled 5% isoflurane (Centro Agrovete Campania, Scafati, SA, Italy) and DNBS (150 mg/kg) was injected in the distal colon using a polyethylene catheter (1 mm in diameter) via the rectum (4.5 cm from the anus). All animals were sacrificed 3 days after DNBS administration by asphyxiation with CO_2_, the mice abdomen was opened by a midline incision and the colon removed, isolated from surrounding tissues, opened along the antimesenteric border, rinsed, weighed, and length measured [in order to determined the colon weight/colon length ratio (mg/cm), used as an indirect marker of inflammation]. Mice body weight was measured every day throughout the treatment period. All measurements were performed by operators who were unaware of the particular treatment (blinded evaluation). For biochemistry analysis, tissues were kept at 80°C until use. The dose of DNBS (150 mg/kg) and the time point of damage evaluation (i.e., 3 days after DNBS administration) were selected on the basis of preliminary experiments showing a remarkable colonic damage associated with high reproducibility and low mortality for this dosage, and because maximal DNBS-induced inflammation has been reported in mice after 3 days (Massa et al., 2004).

### Intestinal Hypermotility Induced by Croton Oil

Increased intestinal motility was induced by the inflammatory agent croton oil as described before (Pol and Puig, 1997; Capasso et al., 2008). Briefly, two doses of croton oil (20 μl/mouse) for two consecutive days were orally administered to mice and 4 days after the first administration of croton oil, upper gastrointestinal transit of mice was measured. This time was selected on the basis of previous work (Pol and Puig, 1997), which reported that the maximal inflammatory response associated to intestinal hypermotility occurs 4 days after the first treatment.

### Upper Gastrointestinal Transit

Upper gastrointestinal transit, measured in control, and in mice with intestinal inflammation-induced accelerated intestinal motility (mice treated with croton oil), was evaluated by identifying the leading front of an intragastrically administered charcoal meal marker (10% charcoal suspension in 5% gum Arabic, 10 ml/kg) in the small intestine as previously described (Capasso et al., 2014). Twenty minutes after charcoal administration, mice were killed by asphyxiation with CO_2_, and the small intestine was isolated by cutting at the pyloric and ileocaecal junctions. The distance traveled by the marker was measured and expressed as a percentage of the total length of the small intestine from pylorus to caecum.

### Pharmacological Treatment

In the experimental model of colitis pure CBD, CBD BDS or vehicle were given intraperitoneally (5–30 mg/kg) or by oral gavage (10–60 mg/kg) for three consecutive days starting 24 h after DNBS administration [Day 0: colitis induction; day 1: CBD BDS (or CBD); day 2: CBD BDS (or CBD); day 3: CBD BDS (or CBD) and mice sacrifice]. The last administration of CBD BDS (or CBD) was given (at day 3) 1 h (for intraperitoneal administration) or 2 h (for oral gavage) before the sacrifice.

In the experimental model of upper gastrointestinal transit pure CBD, CBD BDS, or vehicle were given intraperitoneally (1–10 mg/kg) or by oral gavage (5–60 mg/kg) 30 min (intraperitoneally) or 1 h (oral gavage) before the administration of the marker, to both control mice, and mice with increased intestinal motility induced by the inflammatory agent croton oil. The CBD BDS and pure CBD doses were selected on the basis of previously published work (Borrelli et al., 2009; Romano et al., 2014).

### Myeloperoxidase (MPO) Activity

Myeloperoxidase activity, a peroxidase enzyme used to quantify the neutrophil infiltration in whole-tissue colons, was determined as previously described (Borrelli et al., 2015). Full-thickness colons were homogenized in a lysis buffer composed of 0.5% hexadecyltrimethylammonium bromide in 3-(N morpholino) propanesulfonic acid (MOPS) 10 mM in the ratio of 50 mg tissue per mL MOPS. The homogenates were then centrifuged for 20 min at 15,000 × *g* at 4°C. An aliquot of the supernatant was incubated with sodium phosphate buffer (NaPP pH 5.5) and tetra-methylbenzidine 16 mM. After 5 min, hydrogen peroxide (H_2_O_2_; 9.8 M in NaPP) was added and the reaction stopped with acetic acid. The rate of change in absorbance was measured by a spectrophotometer at 650 nm. Different dilutions of human MPO enzyme of known concentration were used to obtain a standard curve. MPO activity was expressed as U/mg of tissue.

### Tissue CBD Assay: Extraction, Purification, and LC IT-TOF Mass Spectrometry

Colon, liver, and brain samples were dounce-homogenized and extracted with acetone containing internal deuterated standards for CBD quantification by isotope dilution ([^2^H]_4_ CBD). The lipid-containing organic phase was dried down, weighed, and pre-purified by open bed chromatography on silica gel. Fractions were obtained by eluting the column with 99:1, 90:10, and 50:50 (v/v) chloroform/methanol. The 99:1 fraction was used for CBD quantification by LC-MS-IT-TOF analysis using an LC20AB coupled to a hybrid IT-TOF detector (Shimadzu Corporation, Kyoto, Japan) equipped with an ESI interface. We acquired full-scan MS^n^ spectra of selected precursor ions by multiple reaction monitoring (MRM), extracted the chromatograms of the high-resolution [M–H]^-^ values and used the latter chromatograms for calibration and quantification.

#### HPLC Parameters

Liquid chromatography analysis was performed in the isocratic mode using a KinetexC18 column (10 cm × 2.1 mm, I.D. 5 μm, 100 A; Phenomenex) and methanol:water (75:25) with 0.1% NH_4_C_2_H_3_O_2_ as mobile phase with a flow rate of 150 μl/min. The samples were injected with a SIL-20 AC autosampler (Shimadzu Corporation, Kyoto, Japan). The amounts of CBD in tissues, quantified by isotope dilution with the abovementioned deuterated standard, are expressed as ng per mg of tissue weight.

#### Mass Spectrometry Parameters

Electrosprayed ions were generated using a capillary voltage of 4.66 kV. A curved desolvation line (CDL) was set at a temperature of 250°C to aid desolvation and a heat block temperature of 220°C was also used. To help nebulisation of the electrospray solution nitrogen was pumped into the ion source at a rate of 1.5 L/min. The ToF mass analyzer was used to acquire data in both MS and MS/MS modes. In the MS mode, a 10 ms ion accumulation time was used before ion trapping. In the MS–MS mode, instead, the ion accumulation time was 20 ms and the window used for precursor ion isolation corresponds to a width of 3 atomic mass unit (amu) and 20 ms. To induce fragmentation of the precursor ion a supplementary alternative current (AC) potential was applied to the end-cap electrodes to induce resonant excitation and argon is used as a collision gas during collision-induced dissociation (CID). The collision was carried out over 30 ms using a *q*-value of 0.251 (45 kHz). Three scans were accumulated in each MS–MS spectrum. In both MS and MS–MS mode data were acquired over a mass range of 200–500 *m/z*. In both regimes of operation ions are pulsed into the time of flight (ToF) with an accelerating potential of 9 kV and the detector voltage is set at 1.7 kV (Piscitelli et al., 2011).

Full details of the quantification of CBD using LC-MS-IT-ToF-MS will be published elsewhere (Piscitelli et al., 2011, manuscript in preparation].

### Statistical Analysis

Data are expressed as the mean ± SEM of *n* experiments. To determine statistical significance, Student’s *t*-test was used for comparing a single treatment mean with a control mean, and a one-way ANOVA followed by a Tukey–Kramer multiple comparisons test was used for analysis of multiple treatment means. *P*-values < 0.05 were considered significant.

## Results

### Effect of CBD BDS and Pure CBD on Body Weight and Colon Weight/Colon Length *Ratio*

**FIGURE 1:**
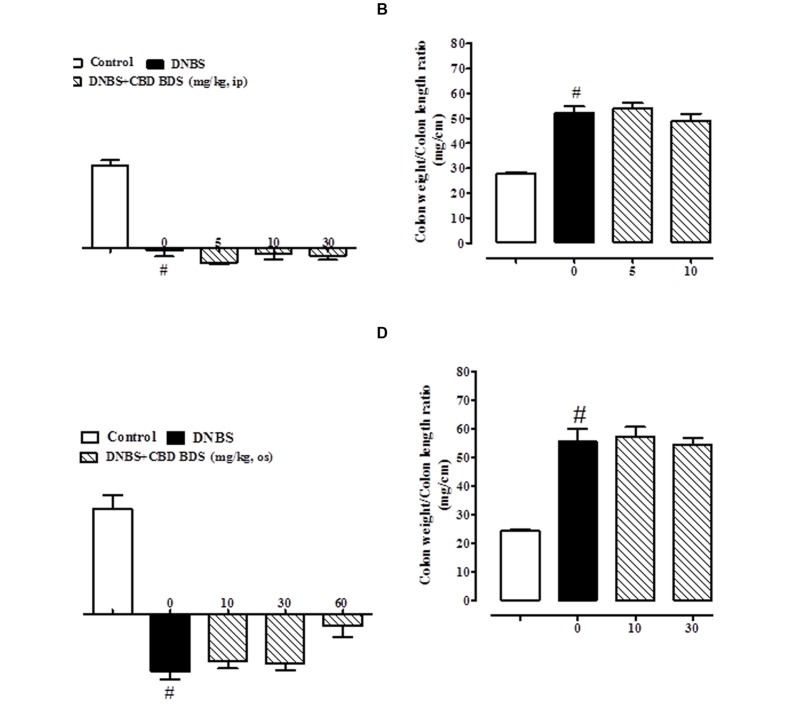
**Effect of CBD BDS (5–30 mg/kg, intraperitoneally) on body weight **(A)** and colon weight/colon length *ratio***(B)** and effect of CBD BDS (10–60 mg/kg, oral gavage) on body weight **(C)** and colon weight/colon length *ratio***(D)** in DNBS induced colitis in mice (DNBS, 150 mg/kg, intracolonically).** CBD BDS was given once a day for three consecutive days starting from 1 day after DNBS administration. Mice were euthanized 3 days after DNBS. Mice were weighted before DNBS (or vehicle) administration and immediately before the sacrifice. Bars are mean ± SEM of 8–12 mice for each experimental group. ^#^*p* < 0.001 vs. control and ^∗^*p* < 0.05 vs. DNBS alone.

**FIGURE 2:**
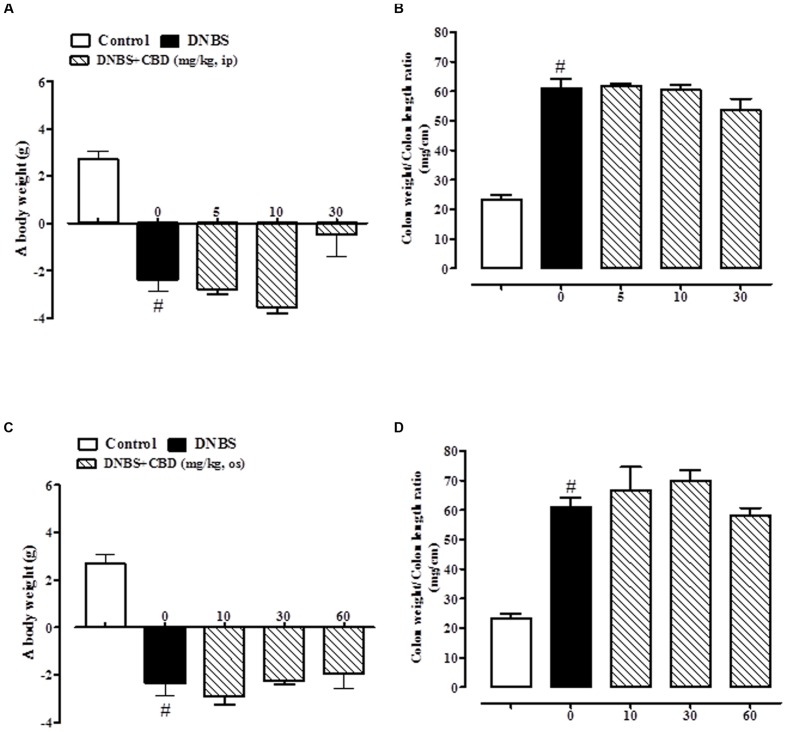
**Effect of pure CBD (5–30 mg/kg, intraperitoneally) on body weight **(A)** and colon weight/colon length *ratio***(B)** and effect of pure CBD (10–60 mg/kg, by oral gavage) on body weight **(C)** and colon weight/colon length *ratio***(D)** in DNBS induced colitis in mice (DNBS, 150 mg/kg, intracolonically).** Pure CBD was given once a day for three consecutive days starting from 1 day after DNBS administration. Mice were euthanized 3 days after DNBS. Mice were weighted before DNBS (or vehicle) administration and immediately before the sacrifice. Bars are mean ± SEM of 8–12 mice for each experimental group. ^#^*p* < 0.001 vs. control.

The administration of DNBS caused both a significant decrease in body weight and a significant increase in colon weight/length *ratio* when compared to control mice (**Figures 1** and **2**). Treatment of DNBS mice with CBD BDS, given intraperitoneally at the range dose of 5–30 mg/kg, did not modify the loss of body weight induced by the inflammatory agent (**Figure 1A**) but reduced DNBS-increased colon weight/length *ratio* (**Figure 1B**). The effect was significant at the 30 mg/kg dose (**Figure 1B**). Likewise, oral gavage administration of CBD BDS (10–60 mg/kg) had no effect on the DNBS-induced decrease in body weight, although a trend in reducing weight loss was observed at the 60 mg/kg dose (**Figure 1C**). CBD BDS, given via oral gavage at the 60 mg/kg dose, significantly reduced the colon weight/length *ratio* increased by DNBS (**Figure 1D**).

Pure CBD, when both given intraperitoneally (5–30 mg/kg) or by oral gavage (10–60 mg/kg), did not ameliorate DNBS-induced colitis (no variation on body weight and colon weight/colon length *ratio*; **Figure 2**).

### Effect of CBD BDS on MPO Activity

**FIGURE 3:**
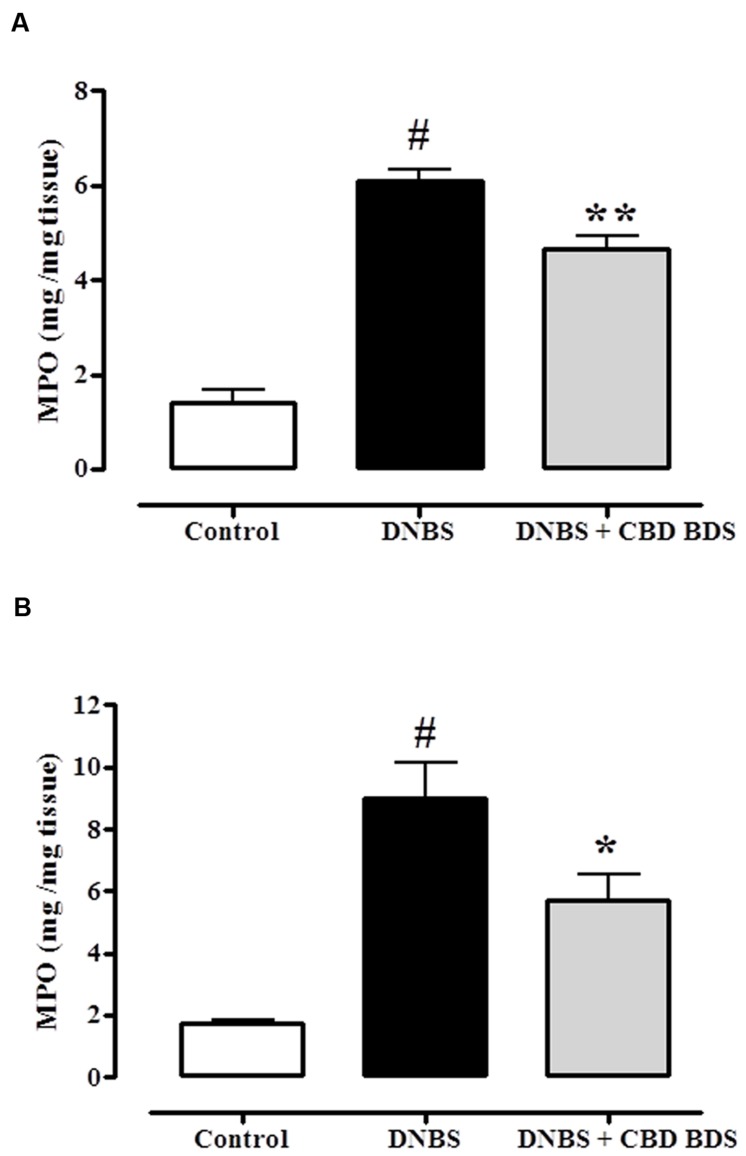
**Inhibitory effect of CBD BDS 30 mg/kg, intraperitoneally **(A)** and CBD BDS 60 mg/kg, by oral gavage **(B)** on myeloperoxidase (MPO) activity in DNBS induced colitis in mice (DNBS, 150 mg/kg, intracolonically).** MPO activity was measured on colonic tissues 3 days after vehicle or DNBS administration. CBD BDS was given once a day for three consecutive days starting from 1 day after DNBS administration. Mice were euthanized 3 days after DNBS. Bars are mean ± SEM of 4–5 mice for each experimental group. ^#^*p* < 0.001 vs. control and ^∗^*p* < 0.05 ^∗∗^*p* < 0.01 vs. DNBS alone.

The beneficial effect of CBD BDS on DNBS-induced colitis was further confirmed by the MPO results. DNBS administration produced a threefold increase in MPO activity which was significantly reduced by either intraperitoneal or oral gavage administration of CBD BDS at the 30 mg/kg and 60 mg/kg dose, respectively (**Figure 3**).

### Effect of Pure CBD and CBD BDS on Upper Gastrointestinal Transit

**FIGURE 4:**
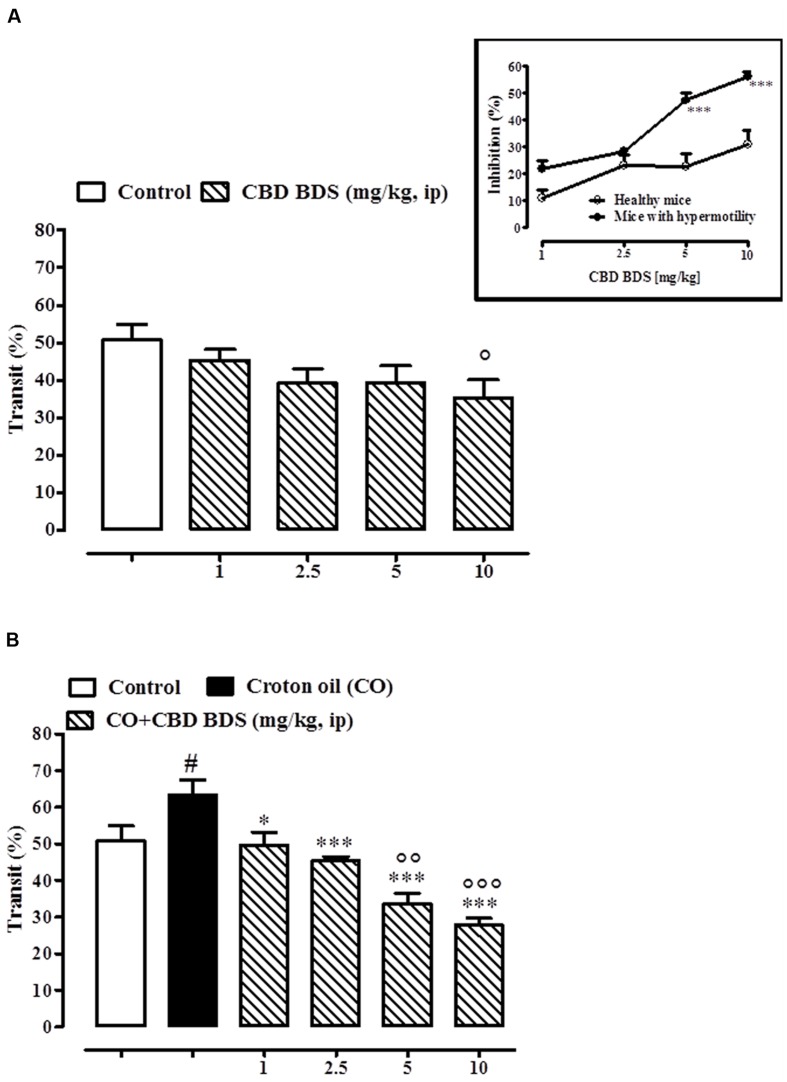
**Effect of CBD BDS (1–10 mg/kg, intraperitoneally) on intestinal transit in healthy mice **(A)** and croton oil-treated mice **(B)**.** Bars represent the mean ± SEM of 8–10 animals for each experimental group. ° and *^#^p* < 0.05, ^∘∘^*p* < 0.01, and ^∘∘∘^*p* < 0.001 vs. control and ^∗^*p* < 0.05 and ^∗∗∗^*p* < 0.001 vs. croton oil alone. Insert: Difference between the curves representing the inhibitory effect of CBD BDS on intestinal transit in healthy mice and mice with hypermotility (mice treated with croton oil). Results are expressed as mean ± SEM of 8–10 mice for each experimental group. ^∗∗∗^*p* < 0.001 vs. healthy mice.

**FIGURE 5:**
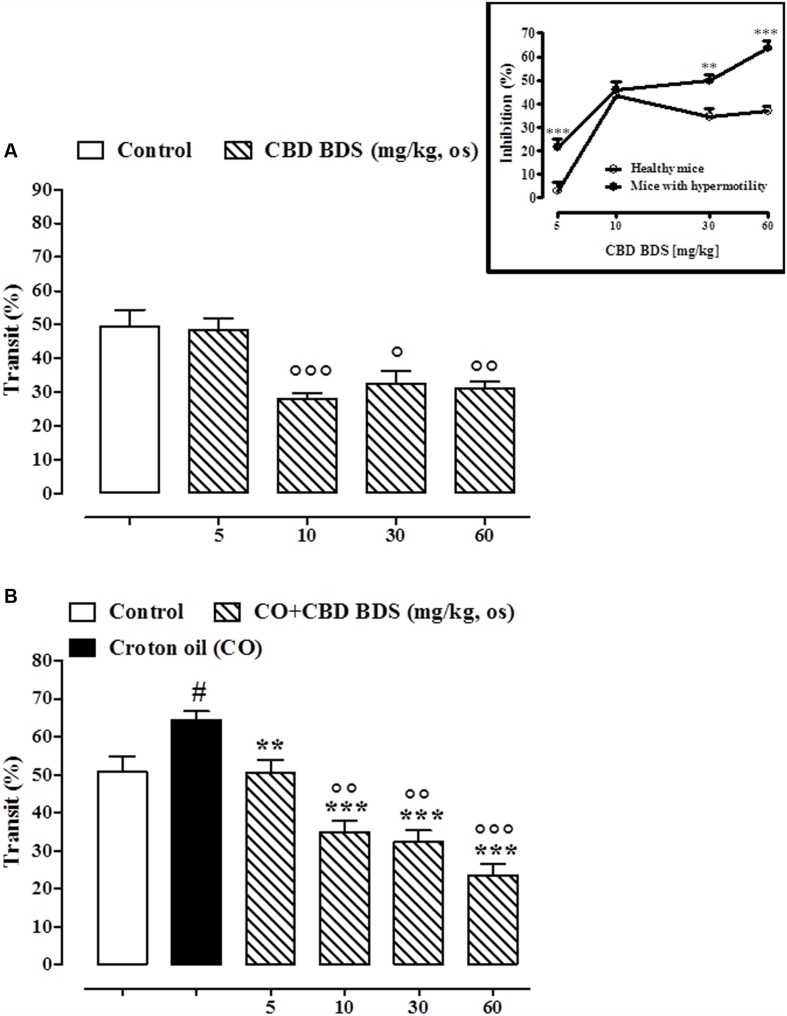
**Effect of CBD BDS (10–60 mg/kg, by oral gavage) on intestinal transit in healthy mice **(A)** and croton oil-treated mice **(B)**.** Bars represent the mean ± SEM of 8–10 animals for each experimental group. ° and ^#^*p* < 0.05, ^∘∘^*p* < 0.01 and ^∘∘∘^*p* < 0.001 vs. control and ^∗∗^*p* < 0.01 and ^∗∗∗^*p* < 0.001 vs. croton oil alone. Insert: Difference between the curves representing the inhibitory effect of CBD BDS on intestinal transit in healthy mice and mice with hypermotility (mice treated with croton oil). Results are expressed as mean ± SEM of 8–10 mice for each experimental group. ^∗∗∗^*p* < 0.001 vs. healthy mice.

CBD BDS, given intraperitoneally at the dosage range of 1–10 mg/kg, reduced the intestinal transit in healthy mice, the effect being significant only at the higher dose tested (10 mg/kg; **Figure 4A**). CBD BDS (1–10 mg/kg), in a dose dependent manner, counteracted the increase in intestinal motility induced by croton oil (**Figure 4B**). The effect was significant starting from the 1 mg/kg dose. Analysis of the curves representing the inhibitory effect of CBD BDS on transit in healthy mice and in mice with hypermotility induced by croton oil shows that CBD BDS preferentially inhibited intestinal transit in pathophysiological rather than physiological conditions (**Figure 4**). Likewise, CBD BDS when given by oral gavage at the dosage range of 5–60 mg/kg reduced the intestinal transit in healthy mice, the effect being significant starting from the 10 mg/kg dose (**Figure 5A**). Similar to the intraperitoneal administration, oral gavage administration of CBD BDS (5–60 mg/kg), in a dose dependent manner, reduced the intestinal hypermotility induced by croton oil (**Figure 5B**). The effect was significant starting from the 5 mg/kg dose. Analysis of the curves representing the inhibitory effect of CBD BDS on transit in healthy mice and in mice with hypermotility induced by croton oil shows that CBD BDS, also when given by oral gavage, preferentially inhibited intestinal transit in pathophysiological rather than physiological conditions (**Figure 5**).

**FIGURE 6:**
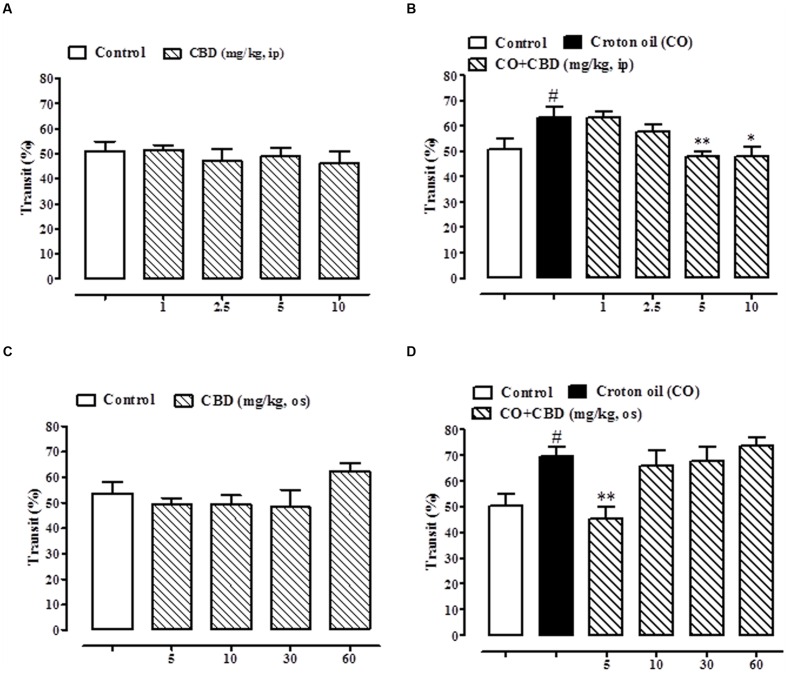
**Effect of intraperitoneal administration of pure CBD (1–10 mg/kg) on intestinal transit in healthy mice **(A)** and croton oil-treated mice **(B)**, and oral gavage administration of pure CBD (5–60 mg/kg) on intestinal transit in healthy mice **(C)** and croton oil-treated mice **(D)**.** Bars represent the mean ± SEM of 8–10 animals for each experimental group. ^#^*p* < 0.05–0.01 vs. control; ^∗^*p* < 0.05 and ^∗∗^*p* < 0.01 vs. croton oil alone.

As previously reported (Capasso et al., 2008), pure CBD, given intraperitoneally at the dose range of 1–10 mg/kg, did not affect the intestinal transit in healthy mice (**Figure 6A**) but, significantly and in a dose dependent manner, restored the intestinal motility in mice with hypermotility induced by croton oil (**Figure 6B**). The effect was significant starting from the 5 mg/kg dose (**Figure 6B**). Oral gavage administration of pure CBD did not reduce intestinal transit in healthy mice (**Figure 6C**), and reduced the croton oil-induced accelerated intestinal motility at the 5 mg/kg dose only (**Figure 6D**).

### CBD Levels in Tissues of Mice with DNBS-Induced Inflammation after Oral Treatment with Pure CBD or CBD BDS

**FIGURE 7:**
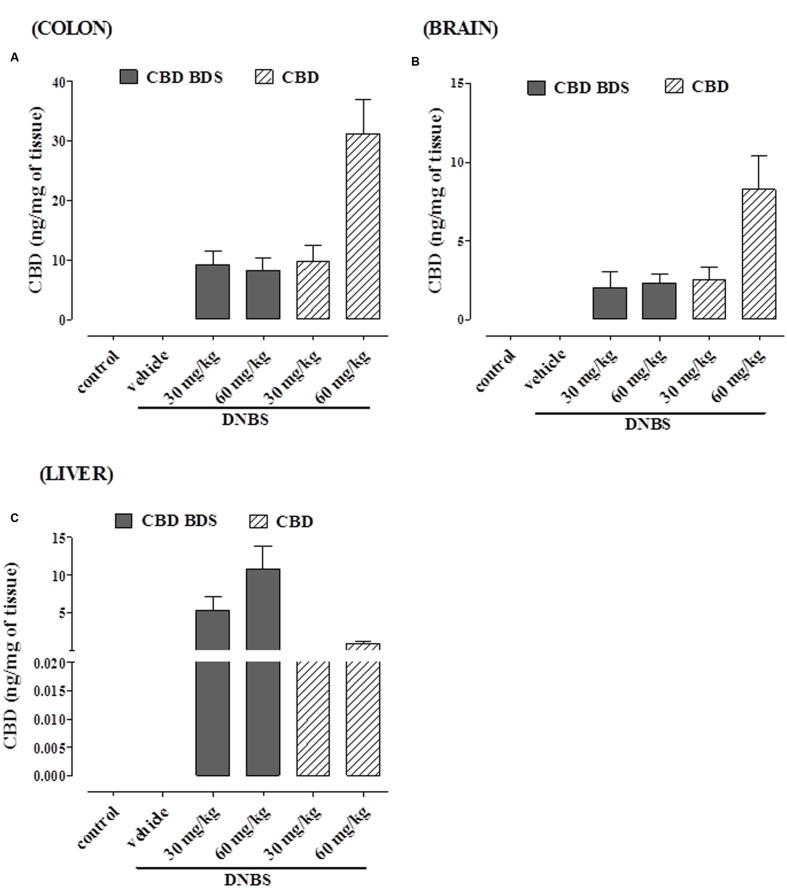
**CBD levels in colon **(A)**, brain **(B)**, and liver **(C)** of DNBS-treated mice after oral administration of either CBD BDS or CBD.** Data are means ± SEM of *N* = 5–6 mice.

As shown in **Figure 7**, CBD was detected in the colon, liver, and brain in mice treated with either CBD BDS or pure CBD (oral administration). In the colon (**Figure 7A**) and in the brain (**Figure 7B**), the content increased with the dose in the case of pure CBD, while in the CBD BDS group there were no differences between the two doses.

In the liver, CBD tissue penetration was higher with the oral administration of CBD-BDS, in a dose-dependent manner (**Figure 7C**), while there were no significant differences between pure CBD at 30 and 60 mg. Interestingly, higher CBD levels were achieved with pure CBD in the colon and brain, and much higher CBD levels were achieved with CBD BDS in the liver.

## Discussion

The notion that not all of the therapeutic effects of *C. sativa* are due to its many active psychotropic ingredient THC is well established (Wilkinson et al., 2003; Russo, 2011; Brodie et al., 2015). The contribution of non-THC phytocannabinoids to *Cannabis* pharmacology has been scientifically demonstrated in a number of experimental diseases, including ulcerative colitis (Duncan and Izzo, 2015). In previous studies, it has been shown that isolated *Cannabis* constituents, including cannabichromene (CBC), CBD and cannabigerol (CBG), exert favorable effects in experimental models of IBD (Borrelli et al., 2009, 2013; Jamontt et al., 2010; Romano et al., 2013). In the present study, we have expanded our knowledge on the intestinal anti-inflammatory effect of phytocannabinoids by showing for the first time that a *Cannabis* extract with high content in CBD, namely CBD BDS, when both given intraperitoneally and by oral gavage, is able to reduce the extent of the damage and to counteract intestinal hypermotility in experimental models of intestinal inflammation. By contrast, we demonstrated that pure CBD, either given intraperitoneally or by oral gavage at matched CBD doses with CBD BDS, after the inflammatory insult does not offer anti-inflammatory effects.

The route of administration for cannabinoids is a major issue since cannabinoids are significantly metabolized by hepatic cytochrome enzymes (Huestis, 2005). There is no evidence in the literature that cannabinoids may exert intestinal anti-inflammatory effects when given orally. While the intraperitoneal route of administration is extensively used in rodent experiments, it appears clear that such a way of delivery is hard to be translated to humans. In the present study, we have shown that CBD BDS, given intraperitoneally or by oral gavage, reduces inflammation associated to DNBS administration. Although we did not provide a microscopic score, as done for CBD in our previous work (Borrelli et al., 2009), the anti-inflammatory effect of CBD BDS was supported by colon length/weight *ratio* and by the MPO measurements, two well-established marker of intestinal inflammation (Krawisz et al., 1984; Kristjánsson et al., 2004). In addition, CBD BDS (intraperitoneally or orally) was pharmacologically active when administered after the inflammatory insult, which is clinically relevant in the light of the observation that the main goal of IBD pharmacotherapy is to cure rather than to prevent. In previous studies aimed at investigating the intestinal anti-inflammatory effects of *Cannabis* and its active ingredients, phytocannabinoids, including CBD, were given before the inflammatory insult (i.e., preventive protocol) (Borrelli et al., 2009; Jamontt et al., 2010; Schicho and Storr, 2012). In contrast to CBD BDS, pure CBD does not exert anti-inflammatory effects either when given intraperitoneally or by oral gavage even with the high doses. The lack of effect of pure CBD (up to 20 mg/kg) after oral gavage, but not after intracolonic, administration has been previous documented (Schicho and Storr, 2012) and may be not surprising if we consider that this type of administration is subjected to a significant first-pass effect (Huestis, 2005).

The difference in efficacy between pure CBD and CBD BDS is likely due to the presence of pharmacologically active ingredients. Indeed, in addition to CBD, CBD BDS contains other phytocannabinoids, such as THC, CBC, and CBG, which have been previously shown to exert anti-inflammatory effects in experimental models of colitis (Jamontt et al., 2010; Borrelli et al., 2013; Romano et al., 2013) and whose colonic levels were not determined in the present study. Furthermore non-cannabinoid *C. sativa* constituents, such as flavonoids, phytosterols, and terpenoids, have been shown to ameliorate murine colitis (Reddy, 1976; Somani et al., 2015; Vezza et al., 2016). On the other hand, the higher efficacy of oral CBD BDS does not seem to be ascribable to higher penetration of CBD in the colon when using this drug. In fact, we show here that the exposure to CBD of both the colon and brain after oral administration of either pure CBD or CBD BDS is higher with the highest dose of pure CBD than with the highest dose of CBD BDS, and similar with the lowest doses of the two drugs. Conversely, in the liver, the concentration of CBD, at both doses tested, was higher with CBD BDS than with pure CBD. Interestingly, the maximal colonic concentrations of CBD at the end of treatment can be calculated to be ∼30 nM with CBD BDS and ∼100 nM with pure CBD, which are not too distant from the potencies of this compound at many of its proposed molecular targets (McPartland and Russo, 2014; Brodie et al., 2015). Others have shown that a non-chemically characterized *Cannabis* extract reduced the severity of rat colitis when administered intracolonically (Wallace et al., 2013).

Inflammatory states in the gut may cause motility disturbances, and alterations in intestinal motility are common debilitating symptoms (Brierley and Linden, 2014). To investigate the effect of CBD BDS on intestinal motility, we adopted the croton oil model of intestinal inflammation-induced hypermotility. This model has been extensively used in the past to evaluate the potential of drugs able to reduce intestinal motility such as opioids (Pol and Puig, 2004) and cannabinoids (Aviello et al., 2008). By using this experimental model, it has been shown that a number of phytocannabinoids, including CBD, CBC, and CBN (Izzo et al., 2000, 2012; Capasso et al., 2008), normalize motility during the inflammatory process, with weak or no effect in control mice. In the present study, we have shown that both CBD BDS and pure CBD, given intraperitoneally or by oral gavage, reduce motility in mice with intestinal inflammation, with weak (CBD BDS) or no (pure CBD) effects in control mice. From a translational viewpoint, the low doses of CBD BDS (and pure CBD) required to normalize motility in the inflamed gut – as well as their pharmacological activity following oral gavage administration – are relevant if we consider that the drugs available to reduce motility may be often associated with constipation (Corsetti and Tack, 2015; Rao et al., 2016).

The anti-inflammatory mode of action of CBD BDS in the gut is still elusive and deserves further *ad hoc* studies. CBD and other minor phytocannabinoids contained in CBD BDS may interact with targets (e.g., components of the so-called endogenous cannabinoid system, TRP channels; De Petrocellis et al., 2011, 2012) which have been linked to IBD (Izzo et al., 2015; Zielińska et al., 2015). Recently, pharmacological blockade of GPR55 has been shown to protect against experimental gut inflammation (Stančić et al., 2015), a relevant finding in the light of the observation that CBD is a GPR55 antagonist (Ryberg et al., 2007; Campos et al., 2012). Conversely, the antioxidant effect is believed to not entirely explain *per se* the gut anti-inflammatory actions exerted by CBD (Esposito et al., 2013).

## Conclusion

The present study reveals for the first time the ability of the CBD BDS to attenuate the severity of inflammation in the DNBS model of colitis, as well as to reduce transit in a model of inflammation-induced dysmotility. The strengths of CBD BDS for a possible clinical use in IBD patients include: (a) its intestinal anti-inflammatory activity following oral gavage administration (in contrast to pure CBD, which was ineffective); (b) its ability to reduce the degree of inflammation in a curative protocol, (c) its ability to reduce motility in the inflamed gut at doses lower than those required to affect motility in control animals. Our results further support the therapeutic rationale for combining CBD with other minor constituents present in *Cannabis sativa*, also in the light of recent positive effects exerted by CBD BDS (also named GWP42003) in IBD patients (Irving et al., 2015).

## Author Contributions

EP performed experiments and was responsible for acquisition, analysis and interpretation of data. BR, SF, and OP performed experiments. FP and AL evaluated endocannabinoid levels. RC, AI, VD, and FB were responsible for conception and design, analysis and interpretation of data and redaction of the manuscript.

## Conflict of Interest Statement

The authors declare that the research was conducted in the absence of any commercial or financial relationships that could be construed as a potential conflict of interest.
